# Allogeneic hematopoietic stem cell transplantation in China: where we are and where to go

**DOI:** 10.1186/1756-8722-5-10

**Published:** 2012-03-18

**Authors:** Meng Lv, Xiao-Jun Huang

**Affiliations:** 1 Peking University People's Hospital, Peking University Institute of Hematology, Beijing Key Laboratory of Hematopoietic Stem Cell Transplantation, No. 11 Xizhimen South Street, Beijng 100044, China

**Keywords:** China, Allo-HSCT, Haploidentical, Relapse, GVHD, Elderly patients

## Abstract

Allogeneic hematopoietic stem cell transplantation (allo-HSCT) is an effective and sometimes the only curative therapy for patients with certain hematological diseases. Allo-HSCT has been practiced in China for approximately 30 years, and great improvements have been made within the past decade, particularly in fields such as the haploidentical HSCT system, strategies to overcome relapse and GVHD, and modified HSCT for elderly patients. This review will describe the current situation and provide a prospective of these unique aspects of Allo-HSCT in China.

## Current status of Allo-HSCT in China

It has been more than thirty years since the first allogeneic bone marrow transplantation was successfully performed in China [1], and during the past decades, substantial progress has been made in the field of allogeneic hematopoietic stem cell transplantation (allo-HSCT).

Currently, there are 104 transplant units certified by China's Ministry of Health and the China Marrow Donor Program. Of these centers, 60% are active and routinely perform HSCT. According to data collected from 50 active centers by the Chinese Hematopoietic Stem Cell Transplantation Registry Group (2007-2011), 5 centers performed more than 100 HSCT cases annually, and approximately 30 centers performed 20-100 cases per year. The total number of HSCT cases in all 50 active centers increased steadily from 1093 cases in 2007 to 1633 cases in 2010 [2]. By the end of 2011, this figure has been greater than 2000 by the preliminary statistics.

**Figure 1:**
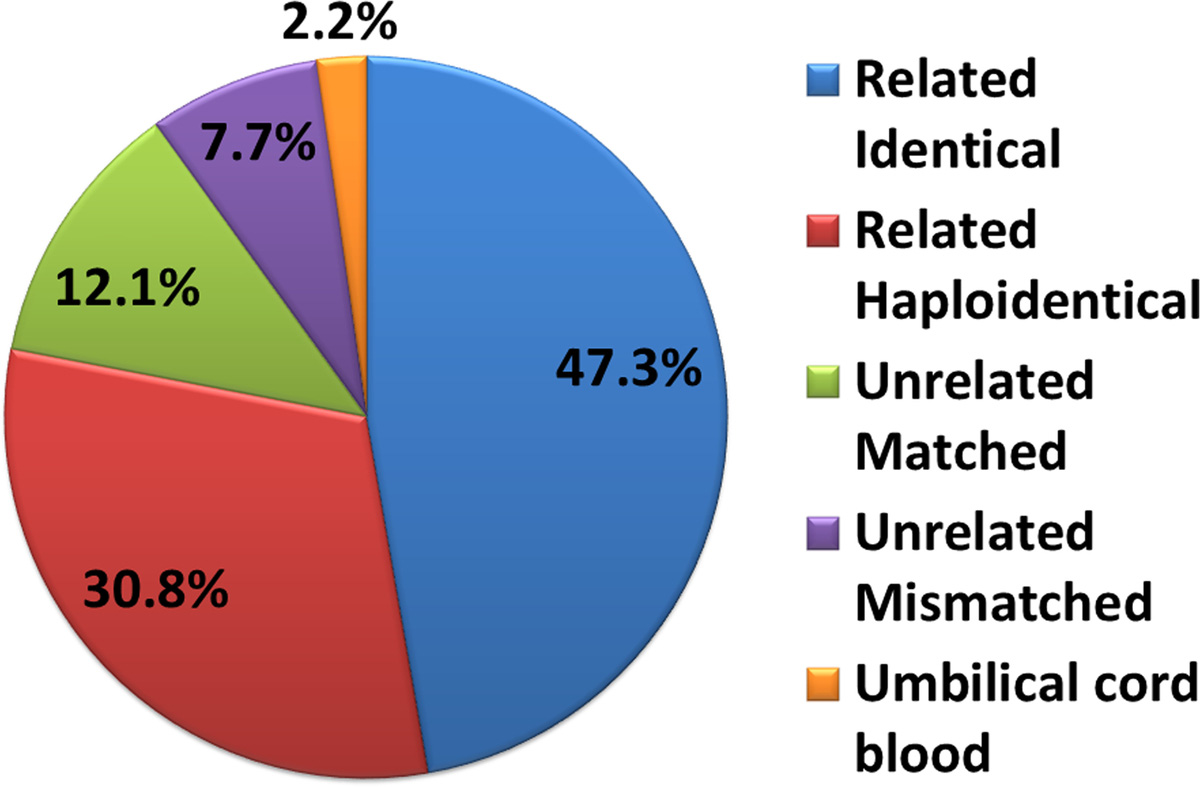
**The types of donor sources for allo-HSCT in China**.

**Figure 2:**
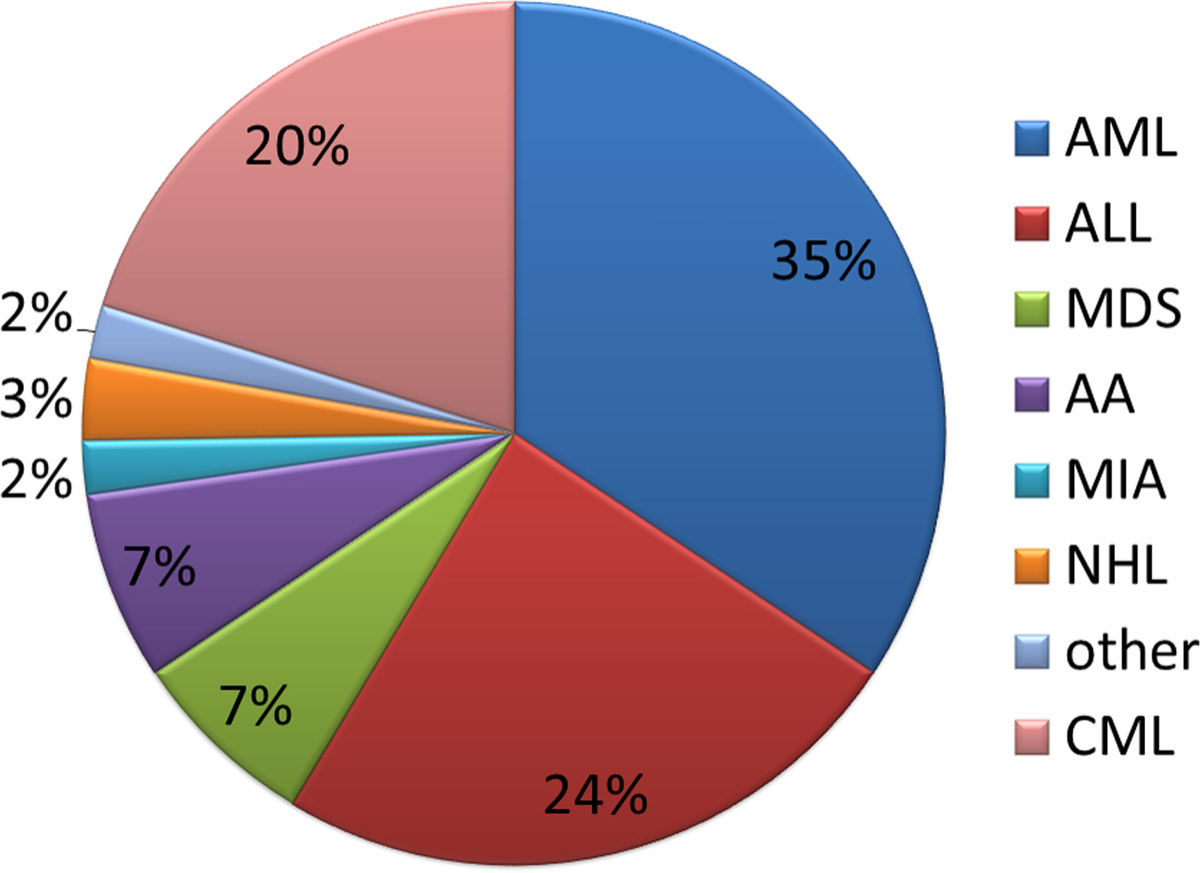
**The distribution of diseases of allo-HSCT recipients in China**.

The types of donor sources for Allo-HSCT in China are related identical (47.3%), related mismatched/haploidentical (30.8%), unrelated matched (12.1%), unrelated mismatched (7.7%), umbilical cord blood (UCB, 2.2%) and Allo-HSCT accounts for 91% of the total HSCT cases. (Figure 1) [2]. These data are quite different from those reported by the CIBMTR(Center for International Blood and Marrow Transplant Research), which show that autologous HSCT takes up 58% of the total HSCT cases and that unrelated donors comprise nearly half of all the allogeneic HSCT graft sources in the USA. Data on haploidentical HSCT(Haplo-HSCT) cases are not available [3]. The distribution of diseases occurring in allogeneic transplant recipients is as follows: acute myeloid leukemia (AML) (34%), acute lymphoblastic leukemia (ALL) (24%), chronic myeloid leukemia (CML) (20%), myelodysplastic syndrome (MDS) (7%), aplastic anemia (AA) (7%), Mediterranean anemia (MIA) (2%), non-Hodgkin's lymphoma (NHL) (3%), and other diseases (2%) (Figure 2). The proportions of disease types receiving allo-HSCT are similar to those reported by the CIBMTR. However, although the overall number of allo-HSCT cases in CML patients has decreased gradually in recent years, these patients still make up a relatively large proportion of the total allo-HSCT cases (approximately 20%). This is due to the potential accumulated expenses of tyrosine kinase inhibitors for young CML patients as well as the significant survival advantage of allo-HSCT compared with imatinimb treatment for patients with AP CML [4].

## The unique characters of Chinese HSCT system

Though research resources for HSCT in China are limited, for example, HSCT researchers are allotted only 16% of the total financial support administered by the National Science Foundation in hematology, an increasing number of clinical and applied fundamental research studies in HSCT have been published in influential journals or presented orally at the American Society of Hematology (ASH) annual meeting. Also, HSCT in China is not just imitation, there are some characteristic aspects which may contribute to other HSCT programs around the world.

These unique characters of Chinese HSCT system can be divided into four categories: ① Haplo-HSCT system, ② strategies to overcome relapse and ③ GVHD, and ④ modified HSCT for elderly patients. The initiation of multi-center clinical trials and advances in translational research are important for promoting progress in HSCT methods in China.

### Evidence-based and mechanism research on Haplo-HSCT

Haplo-HSCT is one of the acceptable alternatives in the absence of HLA-matched siblings or unrelated donors [5,6]. There are three main strategies to complete Haplo-HSCT: T cell-depleted myeloablative HSCT, unmanipulated myeloablative HSCT, or nonmyeloablative/reduced intensity conditioned HSCT [5,7]. Transplantation with T cell-depleted peripheral blood progenitor cells has a low rate of GVHD, but these transplants are associated with slow immune recovery, a high rate of relapse, and a substantial risk of treatment-related mortality [8]. Nonmyeloablative Haplo-HSCT shows relatively lower nonrelapse mortality but a higher rate of overall relapse [9]. The typical Haplo-HSCT systems around the world are summarized in (Table 1) [8-16].

**Table 1:** Studies on Haploidentical Hematopoietic Stem Cell Transplantation

Patients(n)	Disease	Conditioning	Graft/ Manipulation	GVHD Prophylaxis	GF	aGVHD	cGVHD	TRM	LFS/DFS	Nation	Reference
104	AL	ST:TBI/thiotepa/fludarabine/ATG	PB/CD34+ selection	No	1st 6.9%	(II-IV)7.9%	7.1%	36.5%	AL CR:46%-48%	Italy	Aversa (2005)[8]

49	AL/MDS/ CML/MPD	RIC: Flu+Cy/ anti-CD52	PB/anti-CD52	CsA+MMF	6%	(II-IV)16%	14%	10.2%	31-63%@1-3 year	U.S.A	Rizzieri (2007)[10]

24	AL/AA	ST:TBI/CY	BM/costimulatory blockade	CsA+MTX	5%	(III-IV)23.8%	8.3%	50%	33%@7 years	U.S.A	Guinan (2008)[11]

60	AL/NHL/ CML/MM	RIC:Flu/Mel/OKT-3/thiotepa	PB/CD3+CD19 depletion	No if CD3+T < 5 × 104/kg	0	(II-IV)47%	15%	25%@100 days 44%@7 years	HR 41%@1 year 24%@2 years	Germany	Federmann (2009)[12]

66	AL/MDS	RIC:TBI/flu/Bu/ATG/melphalan	PB or BM/No	FK506	6.1%	(II-IV)38%	33.3%	59.1%	28.8%@4 years	Japan	Kurokawa (2010)[13]

820	AL/CML/ NHL/AA	ST:Bu/Cy/Ara-C/MeCCNU/ATG	BM+PB/No	CsA+MTX +MMF	< 1%	(II-IV)42.9% (III-IV)14.0%	Total 53.7% Ex 23.4% @2 years	21%@2 years	SR68.1% HR47.1% @2 years	China	Huang (2011)[14]

83	AL/MDS	RIC:Bu/Flu/ATG	PB/No	CsA+MTX	0	24%	28%	17%@1 year	AML/MDS CR53%-60%; AML RE9%	Korea	Lee (2011)[15]

50	AL/MDS	RIC:Cy/Flu/TBI	BM/No	Cy(HD)+ FK506+MMF	4%	(II-IV)32%	13%	7%@1 year	46%@1 year	U.S.A	Fuchs (2011)[9]

21	AL/CML	ST:Bu/Cy/ MeCCNU/ATG	PB/No	CsA+MTX +MMF	0	(II-IV)33.8%	39.5%	20%@2 years	55.6%@2 years	China	Yu (2012)[16]

*HLA *Human leukocyte antigen; *AL *Acute Leukemia; *CML *chronic myelogenous leukemia; *MDS *myelodysplastic syndrome; *ST *Standard intensity/Myeloablative; *RIC *reduced intensity; *Bu *Busulfan; *Cy *cyclophosphamide; *Ara-C *aracytidine; *TBI *total-body irradiation; *MeCCNU *Semustine; *CsA *cyclosporine A; *MMF *Mycophenolate mofetil; *Flu *fludrabine; *ATG *antithymocyte globulin; *FK506 *Tacrolimus; *anti-CD52 *alemtuzumab; *GF *graft failure; *HR *high risk; *SR *standard risk; *Re *refractory; *Ex *extensive; *GVHD *Graft versus host disease

Due to the "One Child Policy", there has been a shortage of HLA-identical siblings for Chinese patients who need HSCT; what's more, the donor pool of Chinese Marrow Donor Program and cord blood banks are still relatively small in view of the massive Chinese population [17], Therefore haplo-HSCT based on related family donors has a unique role in the treatment of hematologic disease in China. Peking University researchers developed a novel approach for HLA-mismatched/haploidentical myeloablative blood and marrow transplantation without *in vitro *T cell depletion within the past 10 years (the GIAC protocol). This protocol includes the following steps: treating donors with granulocyte colony-stimulating factor (G-CSF) to induce donor immune tolerance, intensified immunological suppression to both promote engraftment and prevent GVHD, antithymocyte globulin (ATG) administration for the prophylaxis of GVHD and graft rejection, and combination of G-CSF-primed bone marrow harvest (G-BM) and G-CSF-mobilized peripheral blood stem cell harvest (G-PB) as the source of stem cell grafts [18-21]. A single-center study reported that unmanipulated myeloablative HSCT using the GIAC protocol has similar LFS/grade, III-IVaGVHD/extensive, chronic GVHD compared with HLA-matched sibling transplantation or matched unrelated donor transplantation [19,21]. The GIAC protocol also demonstrated improved GVL effects [22]. Also, modified haploidentical nonmyeloablative transplantation without T cell depletion and haploidentical PBSCT have been successfully launched as routine procedures in other centers in China [16,23]. Besides Hematological malignancies, haplo-HSCT has become a feasible and safe treatment for nonmalignant hematological diseases, such as severe aplastic anemia [24]. Based on the extensive application, together with unique immunomodulatory strategies for relapse (Referred to 2.2) and mechanism researches, haplo-HSCT of China has developed to a characteristic system. To date, haplo-HSCT accounts for approximately 30% of the total allo-HSCT cases per year in China, and in some experienced centers, such as Peking University Institute of Hematology, this proportion has consistently exceeded 60%, and the cumulative haploidentical cases increased to more than 1300 in single center at the end of 2011.

Though the HLA polymorphism of China is more heterogeneous than that of Japan and Korea, as there are 56 different nationalities in China and the main nationality "Han" also varies in HLA alleles across the vast mainland [25-28]; Haplo-HSCT become more popular in China because of both the well-established clinical regimen and the shortage of sibling and unrelated donors, whereas Japan and Korea marrow donor program have satisfied the need for donors in their countries [26]. What's more, the HLA polymorphism of continental Northeast Asia varies similar to that of Europe and North America [29], which suggest experience of haplo-HSCT in China may be exportable in Europe and North America.

Nevertheless, there are still several problems confronting the future development of haplo-HSCT. First, although the underlying mechanism of crossing the HLA barrier has been partially explained by a series of *in vitro *and *in vivo *studies: ①G-CSF leads to T cell hyporesponsiveness and modulates the balance between Th1 and Th2 immune responses [30]; ②G-CSF treatment significantly decreased the expression of VLA-4, ICAM-1, L-selectin, and LFA-1 on naïve CD4^+ ^and CD8 + T cells in bone marrow grafts. It was also found easier to polarize T cells from Th1 to Th2 in G-BM compared with G-PB [31]; ③T cell hyporesponsiveness and polarization of T cell from Th1 to Th2 could be maintained after in vitro mixture of G-PB and G-BM, besides the benefits of independent component [32]. ④Depletion of infused donor lymphocytes in vivo by ATG, and induction of regulatory T cells [33], etc. However, the complete story has not yet been elucidated. For example, in contrast with ex vivo T cells depletion haplo-HSCT model [34], our finding showed that KIR ligand mismatch is associated with higher aGVHD, a greater relapse rate and inferior survival in our unmanipulated HSCT system [35]; additional results suggested that NK cell allo-reactivity could be covered by massive T cells [36], which need further investigation. Second, reports from single centers are not as convincing as those from multi-center clinical trials. Evaluating a uniform haplo-HSCT protocol, such as GIAC, in more Chinese clinical centers would enable prospective, multi-center trials on the standardized treatment. These types of studies would hopefully aid in evaluating the roles of haplo-HSCT in some uncertain situations.

### Mechanism and management of relapse after HSCT

Relapsing hematologic malignancy after HSCT remains a major cause of death for patients, and finding methods to trigger a more potent graft-versus-tumor effect without increasing the risk of GVHD is still the ultimate goal of HSCT. Many Chinese researchers have attempted to address this challenge.

In a recent study, Huang and his colleagues provide the first report of multiple mutations of CEBPA that contribute to the transformation of donor cells to the leukemic phenotype and provide clues of relapse from donor origin. Huang found that after the first genetic mutation involved in leukemogenesis occurs in the donor, the susceptible donor cells can acquire second or third mutations in different genes or recessive mutations in a single gene after transplantation to the recipient's particular microenvironment. This acquisition of mutations can lead to overt leukemic transformation and cause relapse. This mechanism provides a unique viewpoint that aids our understanding of the process of relapse due to the "multiple genetic-hits mechanism" and suggests that the targeting of mutated genes may be a possible strategy to prevent relapse [37]. Meanwhile, Diamond et al. found relapse of AML after allo-HSCT from donor origin who harbors germline XPD and XRCC3 polymorphisms resulting in low DNA repair capacity [38]. These research together emphasize the importance of gene polymorphisms in selecting candidate donors prior to allo-HSCT.

There is a large gap between laboratory research and clinical application; however, by integrating minimal residual disease (MRD) monitoring and modified donor lymphocyte infusion (mDLI), researchers at Peking University have improved the management of relapse following HSCT in the clinic [39]. Traditional DLI with steady-state lymphocytes has been shown to effectively treat relapse, but it often causes severe GVHD [40]. However, data from other study showed that replacing steady-state lymphocytes with G-PB and applying a short course of immunosuppressive agents (administration of CSA or MTX after DLI for 2-4 weeks) reduced the incidence of DLI-associated acute GVHD without affecting relapse or survival [41]. For patients with hematological relapse after receiving allo-HSCT, the donor GPBPC infusion treatment group had a better outcome compared with chemotherapy; this finding has been demonstrated in both patients under HLA-matched and HLA-mismatched/haploidentical T-cell-replete HSCT groups [42,43]. A later study demonstrated that mDLI treatment also had a prophylactic effect against relapse in high-risk leukemia patients after either of these types of HSCT [44,45]. Recently, our team concluded that risk stratification-directed interventions with mDLI in MRD-positive patients with standard-risk acute leukemia could prevent leukemia relapse and further improve transplant outcomes [46]. Based on these findings, a series of clinical and basic studies were initiated to separate the GVL effect from GVHD with modified DLI in patients with hematological malignancies. These studies found that GPBPC harvests contained more CD34+ cells, Th2-type cells and type II cytokines than non-primed peripheral lymphocyte harvests, which contribute to the lower incidence of aGVHD [32,47]. Meanwhile, the type I cytokines, like IFN-γ, was kept and capable of promoting GVL effects via mechanisms independent of its interaction with leukemia cells [48]. However, the effects of short-course immunosuppressive agents on these lymphocytes remain to be evaluated.

### Prophylaxis and Management of GVHD

GVHD is the main complication post-HSCT, and Chinese doctors must address this condition due to the high proportion of haplo-HSCT cases. Thus, several novel strategies to prevent GVHD have been devised.

The application of low-dose MTX has been demonstrated as a novel regimen for the treatment of GVHD by Peking University research group in different studies. Data from a recent report suggested that MTX in combination with low-dose methylpredinisone was well-tolerated and effective when used as the first-line treatment for aGVHD [49]. It has also been suggested that MTX is a well-tolerated, effective, and inexpensive agent when used as a first-line treatment in combination with other immunosuppressive agents for cGVHD, especially for skin or single organ involvement without concomitant thrombocytopenia [50]. This finding is also supported by a consensus on clinical practices in cGVHD [51].

Lai et al. recently described the efficacy of a new GVHD prophylaxis regimen that combines CSA and MTX with a short 30-day course of low-dose (500 mg/d) mycophenolate mofetil. After allo-HSCT with identical sibling donors, the incidence of acute GVHD was 16% (9.5% for grades 2-4 GVHD and 1% for grades 3 and 4 GVHD). The cumulative incidence of chronic GVHD was 53%, and 28% of these patients experienced the extensive type of disease [52]. Xia et al. reported that basiliximab has a similar effect on aGVHD but superior activity against cGVHD compared with daclizumab [53].

Nevertheless, most of the studies mentioned above are retrospective, and thus, further prospective and multi-center, randomized, controlled studies are needed.

In addition to drugs and antibodies, immunomodulatory cell transfusion may be a more specific method to protect patients from GVHD. Chen et al. reported that heterogenetic bone marrow mesenchymal stem cells (MSCs) could suppress T cell activation. MSC pretreatment was useful in the prevention of GVHD in HLA-mismatched bone marrow transplantation [54]. Xiang et al. reported transfusion of MSCs expanded from the BM of volunteers in vitro was a safe and effective salvage therapy for patients with steroid-resistant cGVHD, which might be mediate by increasing ratio of CD5 + CD19+/CD5-CD19+ regulatory B cells and CD8 + CD28-/CD8 + CD28+ T cells [55]. However, the cotransplantation of MSCs and HSCs may prevent GVHD while elevating the risk of relapse and therefore must be handled with extreme caution.

Basic and Pre-clinical researches on GVHD have also been highly regarded in China. Zhao showed blockade of osteopontin(OPN) could reduce alloreactive CD8+ T cell-mediated GVHD in animal model: OPN promoted the migration and infiltration of naive and alloreactive CD8(+) T cells into host organs; it also facilitated activation and viability of donor-derived CD8(+) T cells via synergizing with CD3 signaling. This findings could validate OPN as a potential target in GVHD prevention [56]. As mentioned above, MMF is an important regimen in controlling GVHD, yet the therapeutic effect of MMF varies in patients. Huang et al. firstly reported the presence of the IMPDH1 IVS8-106 G/G genotype of Inosine monophosphate dehydrogenase (IMPDH, target enzyme of MMF) in recipients was associated with a significantly higher incidence of aGVHD than other genotypes, in both unrelated and sibling transplantation cohorts [57]. This finding would contribute to the individualized therapy of GVHD according to gene polymorphism.

There are also many promising pre-clinical studies focused on the potential application of immune cells in controlling GVHD, such as umbilical cord blood (CB) derived stromal cells [58], ex vivo expanded CB CD4 + CD25+ Foxp 3+ regulatory T cells [59], as well as CXCR4-transduced MSCs, which maintained their immunosuppressive capacity and showed enhanced migration capacity in vitro, thus controlled the occurrence of GVHD more effectively [60].

In short, the progression of these basic and pre-clinical studies would hopefully ameliorate the clinic practices of controlling GVHD in near future.

### Modified HSCT for elderly patients

The outcome of transplantation in patients older than 60 years of age remains unsatisfactory with a low complete remission (CR) rate and poor overall survival (OS) due to prolonged pancytopenia and an elevated chemotherapy-related mortality, etc. [61]. Allogeneic stem cell transplantation after nonmyeloablative or reduced intensity conditioning shows some curative effects for acute leukemia in elderly patients; however, the transplant comorbidities associated with GVHD and infection remain obstacles for this population [62].

Mouse models infused with high doses of G-CSF-mobilized allogeneic spleen cells after cytarabine chemotherapy without immunosuppressive pretreatment exhibited rapid hematopoietic recovery and persistent microchimerism without GVHD. Ai and colleagues designed a clinical control study to show the superiority of "mini-HSCT" over chemotherapy for elderly patients suffering from AML. This HSCT protocol mainly consisted of induction chemotherapy plus the infusion of HLA-mismatched G-PBSCs. Patients who received this treatment had a higher complete remission rate and disease-free survival over 2 years with no observable GVHD. These results indicate that G-PBSCs in combination with conventional chemotherapy may provide a promising treatment method for AML in elderly patients. This finding warrants further multi-center studies to confirm the results and determine if the same method can be applied in the treatment of ALL or other hematopoietic malignancies [63]. Besides, mechanism of "mini-HSCT" remains to be investigated.

## Prospects for the HSCT in China

As mentioned above, multi-center prospective studies and researches connecting "bench to bed" are essential to promote the development of HSCT in China. Actually, there have been successful multi-center trials conducted in general hematology and molecular diagnostics, such as the prospective study "Molecular Diagnosis and Individualized Treatment of Acute Leukemia" supported by the National High Technology Research and Development Program (Program 863). Nevertheless, many challenges remain in the HSCT field: (1) Compared with many well developed international registries, for example, the European Group for Blood and Marrow Transplantation (EBMT), the Chinese Hematopoietic Stem Cell Transplantation Association must make considerable progress in establishing itself as a leader in clinical trials in addition to its role in data management [2]. (2) Standardization would contribute to the improvement and modification of the entire system by allowing results from multiple centers to be compared. However, standardized practices are associated with more precise diagnoses, risk stratification, and corresponding individualized treatment rather than solitary therapies that vary from center to center. The dissemination of techniques is more difficult from experienced centers to smaller HSCT units due to the complexity of the HSCT system, which requires more experienced doctors and diagnostic technologies. (3) Benefit-sharing mechanisms are essential to the success of multi-center clinical trials. A well-developed system would allow all participating units to access data and analyze results from different angles. On the other hand, some experienced centers or institutes have the responsibility to lead clinical research programs applying for financial support, such as the key programs 973 and 863 of China, and to develop well-organized trials.

Next, two strategies can be employed to promote the connection between basic research and clinical practice in the field of Chinese HSCT. Firstly, Chinese researchers must make significant progress in identifying the underlying mechanisms of well-developed clinical models, such as haplo-HSCT. This unique diagnosis and treatment system in turn provides a perfect opportunity to analyze and compare the differences among clinical and animal models. Secondly, translational researchers must pursue promising cellular or animal experiments. For example, researchers from Jilin University, China, found that IFN-γ promotes graft-versus-leukemia effects without directly interacting with leukemia cells in mice after HSCT [48]. Therefore, it would be very interesting to investigate the potential enhancement of GVL effects in DLI or reduced intensity HSCT in patients by manipulation of IFN-γ.

In summary, it would be very meaningful for Chinese scholars to participate in translational researches of clinical significance, such as immune tolerance in HLA-mismatched HSCT, the distinction between GVHD and GVL, and the association between infection and chronic immunologic imbalance.

## Conclusion

China has made substantial progress in the field of allo-HSCT during the past three decades, particularly in the clinical applications and mechanism researches of haplo-HSCT system, strategies to overcome relapse and GVHD, and modified HSCT for elderly patients. With the development of multi-center clinical trials and advances in translational research, these unique fields of allo-HSCT in China will have a promising future and contribute more to the world HSCT system.

## Abbreviations

HSCT: Hematopoietic stem cell transplantation; BMT: bone marrow transplantation; GVHD: Graft versus host disease; CIBMTR: Center for International Blood and Marrow Transplant Research; Allo: Allogeneic; ATG: Antithymocyte globulin; G-BM: G-CSF-primed bone marrow harvest; G-PB: G-CSF-mobilized peripheral blood stem cell harvest; MRD: Minimal residual disease; mDLI: Modified donor lymphocyte infusion; MSCs: Mesenchymal stem cells; CR: Complete remission; OS: Overall survival

## Competing interests

The authors declare that they have no competing interests.

## Authors' contributions

ML collected data and wrote the manuscript, XJH designed and revised the manuscript. All authors read and approved the final manuscript.
